# Perceived offensiveness to the self, not that to others, is a robust positive predictor of support of censoring sexual, alcoholic, and violent media content

**DOI:** 10.3389/fpsyg.2023.1159014

**Published:** 2023-08-29

**Authors:** Jinguang Zhang

**Affiliations:** ^1^School of Journalism and Communication, Sun Yat-sen University, Guangzhou, China; ^2^Center for Big Data and Public Communication, Sun Yat-sen University, Guangzhou, China

**Keywords:** the third-person effect, media censorship, harm, offensiveness, moral foundations

## Abstract

**Introduction:**

Harm and offense are two important notions in legal discussions on the extent to which one’s freedom may be limited. Prior research on the third-person effect found that perceived media harm on others, not perceived media harm on the self, is a robust positive predictor of support of censoring socially undesirable media content (e.g., pornography). In comparison, how offensiveness perceptions predict censorship support is not clear. Drawing on moral foundations theory, we test here how perceived media offensiveness to the self compared with 1) perceived media offensiveness to others and 2) perceived media harm on others would predict censorship support.

**Method:**

We conducted two cross-sectional survey studies in the U.S. to address this question with sexual, alcoholic, and violent media content as test cases. In Study 1 (*N* = 544 undergraduates), we measured perceived media offensiveness to the self, that to others, and censorship support. In Study 2 (*N* = 727 non-student adults), we also measured perceived media harm on the self and others.

**Results:**

As in prior research, we found that people perceive sexual, alcoholic, and violent media content to harm other viewers more strongly than it harms themselves, and the perception of how much others are harmed predicts perceivers’ censorship support. In contrast, while people also perceive the three types of media content to offend other viewers more strongly than they offend the self, the perception of how much others are offended predicts censorship support to a significantly lesser extent or does not predict this at all. Instead, the perception of how much the self is offended does.

**Discussion:**

These findings add to the work on moral foundations theory that distinguishes between how the care/harm and sanctity/degradation foundations relate to moral judgments. These findings also suggest that the current theorizing of the third-person effect needs to expand to reconcile the seemingly inconsistent results on how harm and offensiveness perceptions differently relate to censorship support. The care/harm and sanctity/degradation foundations may underlie how harm and offensiveness perceptions predict censorship support. However, several “anomalous” findings need to be accounted for before moral foundations provide a comprehensive explanation of the third-person effect.

## 1. Introduction

The principles of harm (Mill, 1859/1978) and offense (Feinberg, 1984) frame much of the debate on free speech (Howard, 2019; Bell, 2021). The harm principle argues that the state may limit one’s freedom only to protect others from harm; other than that, the expression of any doctrine—“however immoral it may be considered”—is allowed (Mill, 1859/1978, p. 15). In comparison, the offense principle sets a lower bar for state inference, allowing the regulation of behaviors that cause in others unpleasant emotions such as anger and disgust (i.e., being offensive). In many countries including the U.S., where this research was conducted, the law prohibits the broadcasting on radio and television of content deemed “patently offensive” (The Federal Communications Commission, 2021).

The notions of harm and offense also shape lay people’s views on the extent to which certain media content should be censored (Wilson et al., 1990; Hargrave and Livingstone, 2009). In particular, the large and still growing body of research on the third-person effect (Davison, 1983; Perloff and Shen, 2023) found that the perceived harm of socially undesirable media content (e.g., pornography) on *other* viewers (e.g., rendering them promiscuous)—more so than perceived harm on the self—is a robust positive predictor of censorship support (Chung and Moon, 2016). In comparison, whether the correlation between offensiveness perceptions and censorship support follows the same pattern is unclear. That is, relative to perceived media offensiveness to the self, is perceived media offensiveness to others also a more robust predictor of support of media censorship?

This question is important to address because it concerns whether a basic finding (i.e., the robust positive correlation between perceived media harm on others and censorship support) of a classic phenomenon of media psychology (i.e., the third-person effect) extends to another perception of media effects (i.e., perceived media offensiveness). If it does, the third-person effect should expand to encompass both harm and offensiveness perceptions. Otherwise, an explanation would be needed for why the two perceptions differentially predict censorship support. Either way, we would gain a better understanding of the third-person effect and—more broadly—the intuition underlying the community’s sense of justice (Darley, 2001), in this case, the extent of free speech allowed.

To this end, in this research we examined how perceived media offensiveness to others and to the self would relate to support of censoring sexual, alcoholic, and violent media content drawing on moral foundations theory (Graham et al., 2013). Moral foundations theory is a social psychological theory on the foundations (or constraints) of moral cognitions. The theory posits six such foundations, and two of them—namely, care/harm and sanctity/degradation—closely correspond to the concepts of harm and offensiveness, thus providing a sound theoretical framework for the current research.

We chose sexual, alcoholic, and violent media content as our test cases because they are featured in many media programs (Bleakley et al., 2014; Thrasher et al., 2014), are the focus of media laws and regulations in the U.S. and many other countries, and have been studied extensively in prior third-person effect research (Austin and Meili, 1994; Gunther, 1995; Rojas et al., 1996; McLeod et al., 1997, 2001; Hoffner et al., 1999; Salwen and Dupagne, 1999; Shah et al., 1999; Banning, 2001; Hoffner and Buchanan, 2002; Lo and Wei, 2002; Nathanson et al., 2002; Cho and Han, 2004; David et al., 2004; Lee and Tamborini, 2005; Boyle et al., 2008; Paek et al., 2008; Scharrer and Leone, 2008; Shin and Kim, 2011; Chen et al., 2015; Hong, 2015; Zhang, 2017; Zhou and Zhang, 2023). Thus, using those three types of media content as test cases would render our findings comparable to extant findings while maximizing their theoretical and practical implications.

In what follows, we review the third-person effect in relation to perceptions of media harm and offensiveness, describe moral foundations theory and its prediction of how offensiveness perceptions would predict censorship support, and present findings from two studies that test this prediction.

### 1.1. The third-person effect and perceived media harm and offensiveness

#### 1.1.1. The third-person effect

The third-person effect describes two robust findings. First, people tend to perceive socially undesirable media content to have stronger effects on other viewers (PME3^1^) than on the perceivers themselves (PME1) (Sun et al., 2008). This is known as the third-person perception, which is typically measured as PME3 minus PME1. Second, the intent to act on (e.g., censor) the media content in question tends to increase with third-person perceptions (Xu and Gonzenbach, 2008). This is known as the behavioral component of the third-person effect (henceforth “the third-person effect” for brevity). Chung and Moon (2016) proved that the correct way to test the third-person effect is to regress censorship support on PME3 and PME1 as separate predictors in a regression model, not on their difference term. With this method, Chung and Moon (2016) found that, compared to PME1, PME3 is almost always a more robust predictor of censorship support in 14 extant studies.

#### 1.1.2. Perceived media harm

Most prior research measured perceived media effects as perceived media effects on viewers’ attitudes and behavior (Supplementary Table S1). The attitudinal and behavioral effects of socially undesirable media content are harmful to the extent that viewing those types of media content may induce viewers to accept and perform behaviors undermining their wellbeing. For example, prior research found that viewing pornography positively correlated with having unprotected sex (Tokunaga et al., 2020), and viewing beer commercials positively correlated with the acceptance of excessive drinking (Stautz et al., 2016). Decades of research have also found that exposure to violent media content causes aggressive attitudes and behavioral intent in viewers (Bender et al., 2018). Thus, following prior research (Wilson et al., 1990; Gunther, 1995; Jiang et al., 2021), we call the perceived attitudinal and behavioral effects of socially undesirable media content *perceived media harm*.

#### 1.1.3. Perceived media offensiveness

The emphasis on perceived media harm in prior third-person effect research is understandable as the attitudinal and behavioral outcome of viewing socially undesirable media content has been a major topic of media effects studies (Nabi and Oliver, 2009). However, as Nabi (2009) noted, another “dominant focus of media effects and emotion research has been the emotions that *result* from message exposure” (p. 208, italics are original; see also, Barlett and Gentile, 2010). Prior studies found that media messages are highly effective in evoking emotions in viewers, including anger (Arpan and Nabi, 2011), disgust and fear (Leshner et al., 2009), and offensiveness (Wilson et al., 1990).

Indeed, several studies had endeavored to extend the third-person effect to the “emotional realm” (G. M. Chen and Ng, 2016, p. 182). Neuwirth and Frederick (2002, p. 122) asked participants to rate the influence of news stories on “what the self and others would feel.” Neuwirth et al. (2002, p. 331) asked participants to indicate how much certain news stories would make the self and others “feel negative emotions.” Chen and Ng (2016, p. 184) asked participants to estimate how much online posts on abortion would make the self and others “angry or upset” (p. 184). Especially relevant to this research, Leone (2001) asked participants to report how much sexual and violent media content would irritate and offend the self. Reid et al. (2007) asked participants to report how much pornography would arouse or offend the self and others. Following Leone (2001) and Reid et al. (2007), we call this particular kind of perceived media effect *perceived media offensiveness*.

### 1.2. Moral foundations theory

Moral foundation theory (Graham et al., 2013) starts with the assumption that the human brain contains innate (i.e., organized prior to experience) psychological systems designed by natural selection to solve specific adaptive problems (Barrett and Kurzban, 2006). Because adaptive problems are many in kind (e.g., caring for offspring, detecting cheaters in cooperation, and avoiding pathogens), the psychological systems that had evolved as solutions to those problems are functionally specialized (Barrett and Kurzban, 2006). The functionally specialized psychological systems gave rise to moral cognitions associated with distinct domains of social life. That is, those systems—akin to the foundations for a higher-up edifice—constrain what kind of judgment people would pass on to what kind of actions by the self and others in what areas of social life.

Moral foundations theory posits six highly moralized domains of social life, namely, care/harm, fairness/cheating, loyalty/betrayal, authority/subversion, sanctity/degradation, and liberty/oppression (Iyer et al., 2012; Graham et al., 2013). Most relevant to this research, the care/harm foundation is believed to have co-opted from the motive to protect one’s offspring and is typically activated by cues of others suffering. In comparison, the sanctity/degradation foundation might have evolved from the motive to avoid and neutralize infectious diseases and is typically activated by cues of contamination. When activated, the foundations activate relevant emotions (e.g., anger and disgust), and motivate punishment with the aim of removing the causes of the harm or contamination. Supporting the theory, Koleva et al. (2012) found that the care/harm foundation positively predicted the disapproval of animal testing, death penalty, and using torture in interrogation, whereas the sanctity/degradation foundation positively predicted the disapproval of gambling, casual sex, and same-sex marriage.

### 1.3. Moral foundations theory and The third-person effect

#### 1.3.1. The case of perceived media harm

When making sense of the robust, positive correlation between perceived media harm on *others* and censorship support, prior research formulated an “other-protection” hypothesis. This hypothesis states that an altruistic motive of protecting others from harm drives the support of censoring socially undesirable media content (Rojas et al., 1996; McLeod et al., 2001; Shin and Kim, 2011; Hong, 2015; Rosenthal et al., 2018; Liu et al., 2020; Riedl et al., 2022) and is thus consistent with moral foundations theory, especially regarding the care/harm foundation. Indeed, in Koleva et al. (2012), animal testing, death penalty, and using torture—the three activities whose disapproval the care/harm foundation relates to—all presumably concern third-parties’ (i.e., others’) suffering.

#### 1.3.2. The case of perceived media offensiveness

While moral foundations theory suggests that perceived media harm on others would positively predict censorship support, it suggests that perceived media offensiveness to the *self* would positively predict censorship support. Specifically, an important tenet of moral foundations theory is that moral judgments are often made based on intuition instead of deliberation (Graham et al., 2013). For example, when the sanctity/degradation foundation generates moral judgments, it often does so based on whether an action would “offend” the self or “feel wrong” (Haidt et al., 1993, p. 615).

Consistent with this hypothesis, Zhang (2017) found that the sanctity/degradation foundation—which likely underlies offensiveness perceptions—positively and significantly predicted the support of censoring beer commercials. More relevant to this research, Haidt et al. (1993, p. 617) measured U.S. and Brazilian respondents’ perceptions of offensiveness to self (e.g., “would it bother you?”) and harm on others (e.g., “was anyone hurt?”) regarding a series of victimless actions, including using the national flag to clean a toilet and sibling sex. Haidt et al. (1993) found that perceived offensiveness to the self compared to perceived harm on others was a significantly stronger predictor of support of interference. Similarly, Haidt and Hersh (2001) found that perceived offensiveness to the self but not perceived harm on others positively predicted moral condemnation of anal sex and consensual incest. Extending this offensiveness–harm contrast beyond victimless actions, Miller et al. (2014) found that the perception of how much a harmful action (e.g., hitting someone) upsets the self is a stronger predictor of wrongfulness judgment than the perception of how much the outcome of the action upsets the self. This finding suggests that perceived offensiveness is a more reliable predictor of moral condemnation than perceived harm not only for sexual issues but also for violence.

Indeed, Leone (2001) measured respondents’ perceptions of how sexual and violent movie scenes would offend themselves (e.g., the scenes would make “me” uncomfortable and would irritate and offend “me”) and harm others (e.g., the scenes would render other viewers aggressive) (p. 27–28). Leone (2001) found that perceived media offensiveness to the self but not perceived media harm on others positively correlated with the minimum age limits respondents would set for viewing movies with sexual and violent scenes. This is the only study we know of that explicitly tested how perceived media offensiveness and harm relate to censorship support.

Thus, based on moral foundations theory, the findings reviewed above, and our choice of using sexual, alcoholic, and violent media content as test cases (see Section 1), we first predict that perceived media offensiveness to the self would positively predict the support of censoring sexual (**Prediction 1a**), alcoholic (**Prediction 1b**), and violent (**Prediction 1c**) media content. Drawing on Leone (2001) and Miller et al. (2014), we further predict that perceived media offensiveness to the self would be a stronger predictor than perceived media harm on others of the support of censoring the three types of media content (**Prediction 2a-c**).

**Figure 1:**
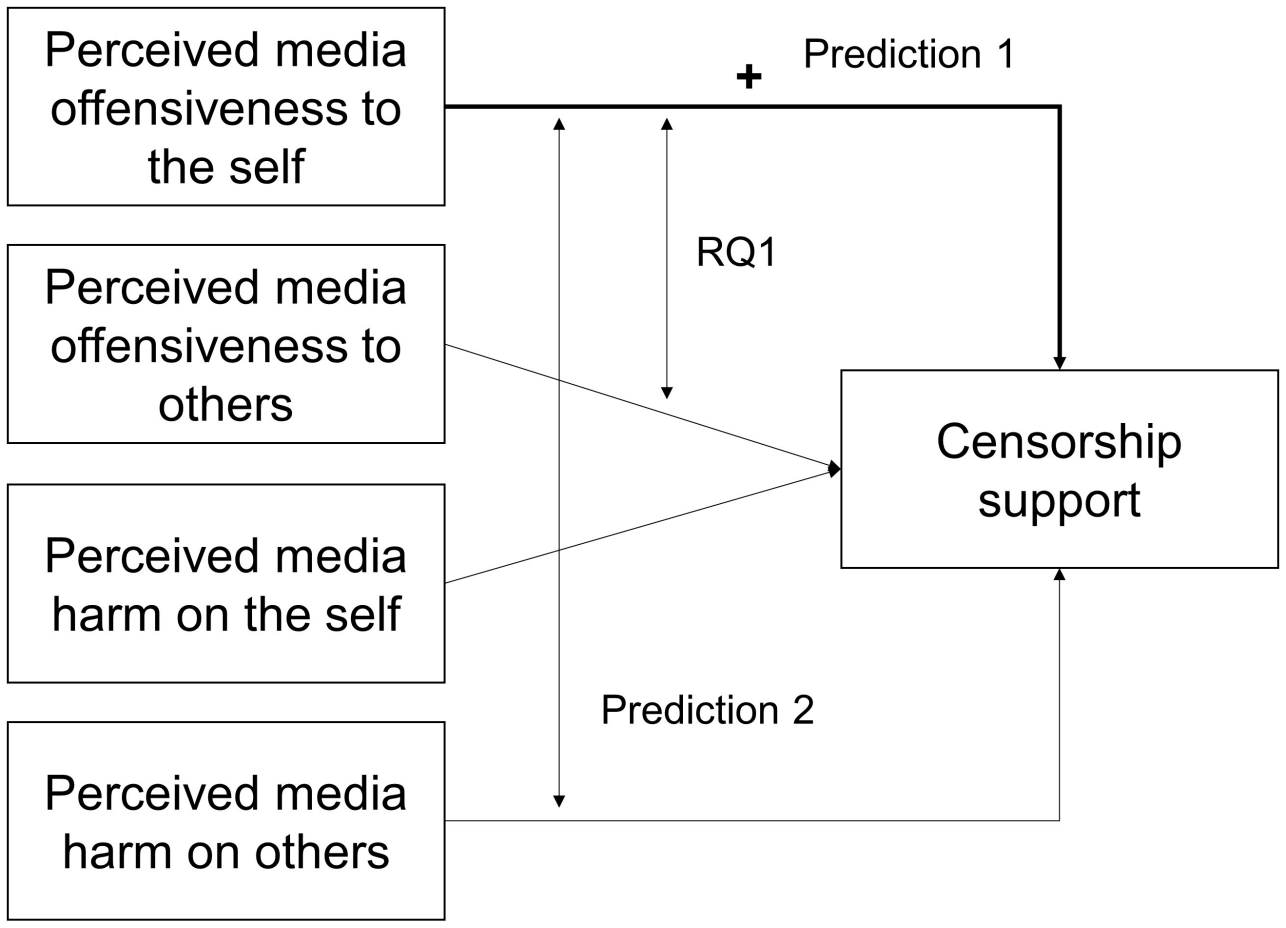
Schematic summary of research predictions and question. The plus sign (+) reads “positively predicts,” the thicker line represents a stronger predictor, and the double-arrowed lines represent contrasts.

Lastly, moral foundations theory did not specify how the perceived offensiveness of an action to *others* would lead to moral judgment, nor did Haidt et al. (1993), Haidt and Hersh (2001), or Leone (2001) include that variable in their studies. We thus had no theoretical or empirical basis to hypothesize the relative predictive power of perceived (media) offensiveness to the self and that to others. We thus left it to a research question (RQ): How would perceived media offensiveness to the self relative to that to others predict censorship support (RQ1)? Figure 1 summarizes our predictions and research question.

### 1.4. Overview of the current research

In Study 1, we measured perceived media offensiveness to the self, that to others, and censorship support with regard to sexual, alcoholic, and violent media content to evaluate Prediction 1 and RQ1. In Study 2, we measured perceived media harm on the self and that on others so that we would be able to evaluate Prediction 2 as well. In both studies, we verified whether we were able to observe third-person perceptions with offensiveness (and harm) perceptions before testing and evaluating our predictions and research question.^2^ To test for third-person perceptions, we performed repeated analysis of variance (ANOVA) followed by simple-effects analyses. To evaluate our predictions and research question, we ran ordinary least square (OLS) regression models followed by the Wald test that compared target regression coefficients.

Recently, Baek et al. (2019) found that PME1 and PME3 interacted to predict the intent to regulate fake news. We thus checked whether our measures of perceived media effects (i.e., the two offensiveness perceptions and the two harm perceptions) interacted to predict censorship support. No interaction effects were significant out of nine tests. See Supplementary Tables S2–S4 for details. We performed all analyses with R (R Core Team, 2023).

## 2. Study 1

### 2.1. Method

#### 2.1.1. Respondents

We recruited *N* = 554 undergraduate students from the University of Hawai‘i at Mānoa to participate in the study in exchange for course credits. We dropped 10 respondents who were reported to be under 18 years old per IRB agreement, leaving us with *N* = 544 for the final sample. The final sample consisted of 56.1% Asian Americans (as is typical of the campus) and 57.4% female students and had a mean age of 20.0 years (SD = 3.84). The protocol of this research was approved by the University of Hawai‘i Institutional Review Board (2018–00002).

#### 2.1.2. Procedure and measures

After completing measures unrelated to this study, respondents read the following prompt: “Certain types of media content are considered offensive; they make one feel uncomfortable, upset, and even annoyed. We would like to know how you feel about the following types of media content in terms of their offensiveness.” The prompt was designed to provide the following items on offensiveness perceptions with a context by defining what being offended meant. Prior research has found that people do find sexual and violent media content offensive (Leone, 2001; Reid et al., 2007) and that the sanctity/degradation foundation—which likely underlies offensiveness perceptions—positively predicts support of censoring beer commercials (Zhang, 2017). If participants did not believe that sexual, alcoholic, and/or violent media content would offend themselves or others, they had the opportunity to express this belief with the scales described below.^3^

After reading the prompt, participants were then asked to indicate with separate items how much they believed that (1) pornography, (2) media portrayals of excessive alcohol consumption, and (3) media violence would offend them (1 *not at all*, 7 *a lot*) (Leone, 2001). Those three questions measured perceived media offensiveness to the self and were presented in random order. Next, respondents were asked to indicate with separate items how much they believed that the same three types of media content would offend other Americans (1 *not at all*, 7 *a lot*). These three questions measured perceived media offensiveness to others and were presented in random order as well. After that, respondents were asked to indicate with separate items how much they would support censoring the three types of media content (1 *strongly oppose*, 7 *strongly support*). Lastly, respondents provided information on their age, sex, and race.

### 2.2. Result

#### 2.2.1. Descriptive statistics and data preparation

Table 1 presents descriptive statistics and inter-correlations.

**Table 1:** Means, standard deviations, and correlations for main variables study 1.

Variable	*M*	SD	1	2	3	4	5	6	7	8
1. Porn self	4.08	2.18								
2. Porn others	4.63	1.84	0.40^**^							
3. Censor porn	4.38	2.17	0.55^**^	0.29^**^						
4. Alcohol self	3.58	1.98	0.65^**^	0.27^**^	0.33^**^					
5. Alcohol others	4.13	1.79	0.34^**^	0.62^**^	0.26^**^	0.44^**^				
6. Censor alcohol	3.96	1.92	0.44^**^	0.26^**^	0.71^**^	0.46^**^	0.32^**^			
7. Violence self	4.96	2.02	0.58^**^	0.21^**^	0.36^**^	0.56^**^	0.28^**^	0.38^**^		
8. Violence others	5.16	1.70	0.26^**^	0.50^**^	0.23^**^	0.24^**^	0.54^**^	0.26^**^	0.43^**^	
9. Censor violence	4.45	2.11	0.38^**^	0.23^**^	0.68^**^	0.32^**^	0.26^**^	0.77^**^	0.42^**^	0.29^**^

Porn = pornography; alcohol = media portrayals of excessive alcohol consumption; and violence = media violence. Porn, alcohol, or violence, self or others = perceived offensiveness of the corresponding content to self or to others. ^**^p < 0.01.

We counted a small number of missing values (48 out of 4,896 total values, or 1%) across the nine main variables (i.e., six offensiveness perception variables and three censorship support variables). There was no evidence that the missing values were differently distributed across the nine variables (ranging 4 to 7 per variable), *χ*^2^ (8) = 1.88, *p* = 0.98. We thus did not impute those missing values.

The absolute values of the skewness of the nine variables ranged from 0.01 to 0.65 (all under 1.5) (Tabachnick and Fidell, 2019), indicating no serious deviation from normality. We thus used the original variables for subsequent analyses. We also recoded sex so that 0 = female and 1 = male and recoded race so that 0 = Asian and 1 = non-Asian, considering that the sample consisted of 56.1% Asian Americans (see Section 2.1.1).

#### 2.2.2. Were there third-person perceptions?

We performed a 2 (target: self/other) × 3 (content: sexual/alcoholic/violent) repeated ANOVA to answer this question. The analysis revealed a significant main effect of target, *F* (1, 536) = 33.4, *p* < 0.001, η^2^_p_ = 0.06, a significant main effect of content, *F* (1, 1072) = 184.4, *p* < 0.001, η^2^_p_ = 0.26, and a significant target × content interaction effect, *F* (2, 1072) = 12.4, *p* < 0.001, η^2^_p_ = 0.02. The significant main effect of target indicated a significant third-person perception averaged across media content. Simple-effect analyses further showed that the third-person perception was significant with all three kinds of media content: pornography, *t* (536) = 5.74, *p* < 0.001, Cohen’s *d* = 0.23; media portrayals of excessive drinking, *t* (536) = 6.50, *p* < 0.001, Cohen’s *d* = 0.27, and with media violence as well, *t* (536) = 2.14, *p* = 0.03 Cohen’s *d* = 0.10. Relevant means and standard deviations are demonstrated in Table 1.

#### 2.2.3. Did perceived offensiveness to the self positively predict censorship support?

Prediction 1 stated that perceived media offensiveness to the self would positively predict censorship support. To test this prediction, we ran three OLS regression models—one for each type of media content—predicting censorship support from the two offensiveness perceptions and respondents’ sex, age, and race. We controlled for sex because prior research found that female relative to male respondents generally were more supportive of censoring sexual (Lo and Wei, 2002), alcoholic (Zhang, 2017), and violent (Hong, 2015) media content. We controlled for the two other demographic variables to explore their potential effects.

The results are summarized in Table 2. As predicted, perceived media offensiveness to the self positively and significantly predicted support of censoring all three types of media content (Table 2). Perceived offensiveness to others positively and significantly predicted censorship support with media portrayals of excessive drinking and media violence.

**Table 2:** OLS regression results of predicting censorship support from offensiveness perceptions study 1.

	*Outcome variable: Support of censoring…*		
	Pornography	Media portrayals of excessive drinking	Media violence
Perceived offensiveness to the self	0.45^***^	0.36^***^	0.37^***^
	(0.05)	(0.05)	(0.05)
Perceived offensiveness to others	0.09^*^	0.16^***^	0.13^**^
	(0.05)	(0.05)	(0.06)
Sex (female = 0, male = 1)	−0.41^**^	−0.26	−0.48^**^
	(0.20)	(0.17)	(0.19)
Age	−0.04^*^	−0.04^*^	0.02
	(0.02)	(0.02)	(0.02)
Race (Asian = 0, non-Asian = 1)	−0.34^*^	−0.40^**^	−0.33^*^
	(0.18)	(0.17)	(0.19)
Constant	3.27^***^	3.01^***^	1.91^***^
	(0.53)	(0.45)	(0.58)
*N*	447	448	447
*R* ^2^	0.31	0.25	0.21
Adjusted *R*^2^	0.30	0.24	0.20
*F*	39.6^***^	29.5^***^	23.6^***^

Standard errors are in parentheses. ^*^p < 0.05; ^**^p < 0.01; ^***^p < 0.001.

#### 2.2.4. Perceived media offensiveness to the self vs. that to others as predictors

RQ1 asked how perceived media offensiveness to the self compared with perceived media offensiveness to others would predict censorship support. We noted from Table 2 that the effect sizes of perceived media offensiveness to the self were 5.0, 2.2, and 2.8 times as large as those of perceived media offensiveness to others of pornography, media portrayals of excessing drinking, and media violence. To confirm that perceived media offensiveness to the self was the stronger predictor of censorship support, we performed the Wald test, constructed as below:


$$Wald\;Z=\left(\beta_{1}-\beta_{2}\right)/SE_{\beta_{1}-\beta_{2}}$$

In the equation above, $\beta_{1}$ and $\beta_{2}$ are the two regression coefficients to be compared, and $SE_{\beta_{1}-\beta_{2}}$ is the standard error of the difference between the two coefficients and given by:


$$SE_{\beta_{1}-\beta_{2}}=\sqrt{Var\left(\beta_{1}\right)+Var\left(\beta_{2}\right)-2\mathrm{cov}\left(\beta_{1},\beta_{2}\right)}$$

In the equation above, *Var* represents variance, and *Cov* represents covariance. The Wald test showed that the coefficient of perceived media offensiveness to the self was significantly larger than that of perceived media offensiveness to others: for pornography, *Z* = 4.38, *p* < 0.001; for media portrayals of excessive drinking, *Z* = 2.38, *p* = 0.02; and for media violence, *Z* = 2.58, *p* = 0.01. Because RQ1 was exploratory in nature, we applied the Bonferroni method to correct for potential familywise Type-1 errors. The contrast was no longer significant for media portrayals of excessing drinking (*p* = 0.06) but remained significant for pornography and media violence (*p* < 0.001 and = 0.03).

### 2.3. Discussion

In Study 1, we observed third-person perceptions with offensiveness perceptions for sexual, alcoholic, and violent media content. Respondents on average perceived pornography, media portrayals of excessive drinking, and media violence to offend others more strongly than offend the perceivers themselves. However, perceived media offensiveness to the self more so than perceived media offensiveness to others positively and significantly predicted support of censoring the three types of media content. Perceived media offensiveness to others did not significantly predict support of censoring pornography, and the effect size of perceived media offensiveness to the self was at least twice as large as that of perceived media offensiveness to others. Those differences were significant with pornography and media violence even after we applied one of the most conservative methods to control for false positive findings. Thus, the findings of Study 1 supported Prediction 1 and addressed RQ1.

As a limitation, we did not measure media harm perceptions, which may correlate with media offensiveness perceptions. Thus, a more stringent test of our prediction may require controlling for harm perceptions. Second, Study 1 used an undergraduate sample, which limited the external validity of its findings. We addressed those two limitations in Study 2.

## 3. Study 2

In Study 2, we tested both Prediction 1 (i.e., the positive correlation between perceived media offensiveness to the self and censorship support) and Prediction 2 (i.e., perceived media offensiveness to the self being a stronger predictor than perceived media harm on others of censorship support) and evaluated RQ1.

### 3.1. Method

#### 3.1.1. Respondents

We recruited *N* = 750 U.S. MTurk workers to participate in this study for a small payment. A simulation-based power analysis (Arend and Schäfer, 2019) indicated that this sample size almost guaranteed us to find a significant contrast between perceived media offensiveness to the self vs. that to others as per RQ1.^4^ To ensure data quality, we required that all workers had a HIT approval rate greater than 98% and more than 1,000 approved HITs (e.g., http://datacolada.org/92). We dropped 23 respondents who had participated in our prior studies, leaving *N* = 727. This final sample consisted of 78.1% non-Hispanic whites and 53.5% male people, and they had a median age of 39 years. The research protocol was approved by the University of Hawai‘i Institutional Review Board (2020–00973).

#### 3.1.2. Procedure and measures

All respondents were assigned to two blocks of questions in random order. In the block on perceived media offensiveness, respondents first read the following prompt: **“**Certain media content are considered offensive, causing displeasure, resentment, and hurt feelings in viewers. What do you think of the following types of media content?” Next, respondents were asked to indicate with separate items how much they believed pornography would offend (1) them and (2) most other Americans (1 *not at all*, 7 *very much*). The two questions were then repeated with “beer commercials that encourage alcohol consumption” replacing “pornography” and then with “graphic violence.”

In the block on perceived media harm, respondents first read the following prompt: “Certain media content is considered effective in changing viewers’ behavior. What do you think of the following types of media content?” Next, respondents were asked to indicate with separate items how likely they believed that viewing pornography would make (1) them and (2) most other Americans develop sexually promiscuous behaviors (1 *not likely at all*, 7 *very likely*) (Lo and Wei, 2002). Respondents were then asked to indicate with separate items how likely they believed that viewing beer commercials would make them and most other Americans develop binge drinking behaviors (1 *not likely at all*, 7 *very likely*) (Zhang, 2017). Finally, respondents were asked to indicate with separate items how likely they believed that viewing graphic violence would make them and most other Americans develop violent behaviors (1 *not likely at all*, 7 *very likely*) (Hoffner et al., 1999).

After that, all respondents were asked with separate items to indicate how much they would support censoring (1) pornography, (2) beer commercials, and (3) graphic violence (1 *do not support at all*, 7 *support very much*) before providing demographic information on their age, sex, ethnicity, and race.

### 3.2. Results

#### 3.2.1. Descriptive statistics and data preparation

Tables 3–5 present descriptive statistics and zero-order inter-correlations.

**Table 3:** Means, standard deviations, and correlations for variables on pornography study 2.

Variable	*M*	SD	1	2	3	4
1. Perceived offensiveness to self (pornography)	3.27	2.16				
2. Perceived offensiveness to others (pornography)	4.12	1.38	0.44^**^			
3. Perceived harm on self (pornography)	3.00	1.91	0.36^**^	0.24^**^		
4. Perceived harm on others (pornography)	3.78	1.79	0.42^**^	0.24^**^	0.77^**^	
5. Censorship support (pornography)	2.98	2.18	0.73^**^	0.30^**^	0.38^**^	0.42^**^

^*^p < 0.05; ^**^p < 0.01.

**Table 4:** Means, standard deviations, and correlations of variables on beer commercials study 2.

Variable	*M*	SD	1	2	3	4
1. Perceived offensiveness to self (beer commercials)	2.60	1.89				
2. Perceived offensiveness to others (beer commercials)	2.73	1.47	0.64^**^			
3. Perceived harm on self (beer commercials)	2.50	1.80	0.54^**^	0.54^**^		
4. Perceived harm on others (beer commercials)	3.32	1.74	0.56^**^	0.47^**^	0.74^**^	
5. Censorship support (beer commercials)	2.69	1.93	0.68^**^	0.49^**^	0.48^**^	0.52^**^

^*^p < 0.05; ^**^p < 0.01.

**Table 5:** Means, standard deviations, and correlations of variables on graphic violence study 2.

Variable	*M*	SD	1	2	3	4
1. Perceived offensiveness to self (graphic violence)	3.84	1.99				
2. Perceived offensiveness to others (graphic violence)	4.19	1.39	0.49^**^			
3. Perceived harm on self (graphic violence)	2.51	1.76	0.35^**^	0.26^**^		
4. Perceived harm on others (graphic violence)	3.30	1.67	0.42^**^	0.32^**^	0.78^**^	
5. Censorship support (graphic violence)	3.42	2.11	0.64^**^	0.35^**^	0.39^**^	0.47^**^

^*^p < 0.05; ^**^p < 0.01.

We counted a small number of missing values (47 out of 10,905 total values, or 0.4%) across the 15 main variables (i.e., 6 offensiveness perception variables, 6 harm perception variables, and 3 censorship support variables). The missing values did not differently distribute across the 9 variables, *χ*^2^ (15) = 2.36, *p* = 0.99. We thus did not impute those missing values.

The absolute values of the skewness of the 9 variables ranged from 0.01 to 0.96 (all under 1.5), and we thus used the original variables for subsequent analyses. We recoded sex so that 0 = female and 1 = male and recoded race so that 0 = non-Hispanic whites and 1 = other, considering that the sample consisted of 78.1% non-Hispanic whites (see Section 3.1.1).

#### 3.2.2. Were there third-person perceptions?

We addressed this question by performing a 2 (target: self/other) × 3 (content: pornography/beer commercials/graphic violence) × 2 (type of effect: offensiveness/harm) repeated ANOVA. All effects in this analysis were significant, including the main effect of target, *F* (1, 721) = 401.0, *p* < 0.001, η^2^_p_ = 0.36. This main effect indicated a significant third-person perception averaged across media content and types of effect. Importantly, the third-person perception was significant for each combination of media content and types of effect.

Specifically, respondents perceived significantly stronger offensiveness to others than to the self for pornography, *t* (721) = 11.5, *p* < 0.001, Cohen’s *d* = 0.43; beer commercials, *t* = 2.57, *p* = 0.01, Cohen’s *d* = 0.09; and graphic violence, *t* = 5.51, *p* < 0.001, Cohen’s *d* = 0.20. Respondents also perceived significantly stronger harm on others than on the self for pornography, *t* = 16.4, *p* < 0.001, Cohen’s *d* = 0.62; beer commercials, *t* = 17.2, *p* < 0.001, Cohen’s *d* = 0.64; and graphic violence, *t* = 18.4, *p* < 0.001, Cohen’s *d* = 0.69. Relevant means and standard deviations are in Tables 3–5.

#### 3.2.3. Did perceived offensiveness to the self positively predict censorship support?

Prediction 1 states that perceived media offensiveness to the self would positively predict censorship support. To test this prediction, we ran three OLS regression models—one for each type of media content—predicting censorship support from the two media offensiveness perceptions, the two media harm perceptions, and respondents’ age, sex, and race. The results are summarized in Table 6.

**Table 6:** OLS regression results of predicting censorship support from harm and offensiveness perceptions study 2.

	*Outcome variable: Support of censoring…*		
	Pornography	Beer commercials	Graphic violence
Perceived offensiveness to the self	0.67^***^	0.51^***^	0.55^***^
	(0.03)	(0.04)	(0.04)
Perceived offensiveness to others	−0.04	0.07	0.02
	(0.05)	(0.05)	(0.052)
Perceived harm on the self	0.09^*^	0.03	0.03
	(0.05)	(0.05)	(0.06)
Perceived harm on others	0.11^**^	0.21^***^	0.30^***^
	(0.05)	(0.05)	(0.06)
Sex (female = 0, male = 1)	−0.35^***^	−0.38^***^	−0.36^***^
	(0.12)	(0.11)	(0.13)
Age	−0.00	−0.00	−0.01
	(0.01)	(0.00)	(0.01)
Race	0.24	0.07	−0.17
	(0.15)	(0.14)	(0.16)
Constant	0.60^**^	0.69^***^	0.60^*^
	(0.29)	(0.25)	(0.31)
*N*	672	670	669
*R* ^2^	0.56	0.49	0.47
Adjusted *R*^2^	0.56	0.49	0.46
*F*	120.8^***^	91.6^***^	82.9^***^

Race (non-Hispanic whites = 0, other = 1); standard errors are in parentheses. ^*^p < 0.05; ^**^p < 0.01; ^***^p < 0.001.

Supporting Prediction 1, perceived media offensiveness to the self positively and significantly predicted support of censoring pornography, beer commercials, and graphic violence, whereas perceived media offensiveness to others did not predict any censorship support. In contrast, perceived media harm on the self did not predict any censorship support, but perceived media harm on others positively and significantly predicted censorship support for all three types of media content.

#### 3.2.4. Perceived media offensiveness to the self vs. perceived media harm on others

Prediction 2 states that perceived media offensiveness to the self would be a stronger predictor of censorship support than perceived media harm on others would be. We tested this prediction as we tested Prediction 2 in Section 3.2.4. As predicted, the effect size of perceived media offensiveness to the self was significantly stronger than that of perceived media harm on others: for pornography, *Z* = 8.61, *p* < 0.001; for beer commercials, *Z* = 4.29, *p* < 0.001; and for graphic violence, *Z* = 3.30, *p* < 0.001. The relevant regression coefficients are demonstrated in Table 6.

#### 3.2.5. Perceived media offensiveness to the self vs. that to others

RQ1 asks whether perceived media offensiveness to the self would be a stronger predictor than perceived media offensiveness to others of censorship support. We performed three Wald tests—one for each type of media content—to address this question. As in Study 1, the effect size of perceived media offensiveness to the self was significantly larger than that of perceived media offensiveness to others: for pornography, *Z* = 10.9, *p* < 0.001; for beer commercials, *Z* = 5.84, *p* < 0.001; and for graphic violence, *Z* = 6.96, *p* < 0.001. These contrasts remained significant after we applied the conservative Bonferroni method to adjust for Type-1 error rates. The relevant regression coefficients are demonstrated in Table 6.

#### 3.2.6. Perceived media harm on the self vs. perceived media harm on others

Lastly, we compared the relative predictive power of perceived media harm on the self and that on others as an attempt to replicate Chung and Moon (2016). Consistent with Chung and Moon (2016), perceived media harm on others positively and significantly predicted the support of censoring all three kinds of media content, whereas perceived media harm on the self did not. The Wald test confirmed that the contrast between perceived media harm on others and that on the self was significant for beer commercials (*Z* = 2.15, *p* = 0.03) and graphic violence (*Z* = 2.48, *p* = 0.01). The contrast for pornography was not.

### 3.3. Discussion

In Study 2, we first observed significant third-person perceptions for both media offensiveness and harm perceptions and each type of media content (i.e., pornography, beer commercials, and graphic violence). Second, as in prior research (Chung and Moon, 2016), perceived media harm on others but not that on the self significantly and positively predicted the support of censoring the three types of media content. Importantly, we replicated Study 1 that perceived media offensiveness to the self—not perceived media offensiveness to others—that positively and significantly predicted the support of censoring all three types of media content. Further, the effect size of perceived media offensiveness to the self was statistically stronger than that of perceived media offensiveness to others. Lastly, we corroborated Leone (2001) that perceived media offensiveness to the self was a significantly stronger predictor than perceived media harm on others of the support of censoring pornography, beer commercials, and graphic violence. Our findings supported all three predictions.

## 4. General discussion

In two studies, we explored how media offensiveness perceptions predicted support of censoring sexual, alcoholic, and violent media content drawing on moral foundations theory with two diverse samples of U.S. adults. First, we found significant third-person perceptions with both offensiveness and harm perceptions for each type of media content. These results corroborated prior work on the third-person perception of emotional media effects (Neuwirth et al., 2002; Neuwirth and Frederick, 2002; Reid et al., 2007; Chen and Ng, 2016), indicating that perceived media offensiveness is a valid measure of perceived media effects.

Second, we found that perceived media harm on others (i.e., a measure of PME3) relative to perceived media harm on the self (i.e., a measure of PME1) was the more reliable predictor of censorship support. This finding is consistent with prior research that focused on the perceived effects of socially undesirable media content on viewers’ attitudes and behavior (Chung and Moon, 2016). In other words, we replicated the classic third-person effect with perceptions of media harm.

Importantly, we found in both studies of this research that perceived media offensiveness to the self (i.e., a measure of PME1) positively and significantly predicted censorship support and it did so significantly more strongly than perceived media offensiveness to others (i.e., a measure of PME3). In addition, perceived media offensiveness to others only significantly predicted censorship support in two out of six tests across the two studies. These findings reversed the third-person effect, where PME3—not PME1—is typically the more reliable predictor of censorship support.

Perhaps equally notable is the finding that perceived media offensiveness to the self is also a stronger predictor than perceived media harm on others of the support of censoring sexual, alcoholic, and violent media content. This finding corroborated that of Leone (2001) and suggests that perceived media offensiveness to the self plays a more important role than perceived media harm on others in regulating people’s support of censoring the three types of media content tested in this research. Collectively, the findings of this research have important implications for moral foundations theory and theorizing the third-person effect, and we discuss them in the following sections.

### 4.1. Implications for moral foundations theory

Moral foundations theory (Graham et al., 2013) made—and subsequent research (Haidt et al., 1993; Haidt and Hersh, 2001; Koleva et al., 2012; Miller et al., 2014) verified—a clear distinction between the roles of harming others and offending the self in people making moral judgments. In other words, the care/harm foundation and the sanctity/degradation foundation appear to focus on different targets (i.e., others vs. the self) when motivating moral judgment. However, we know of no evidence that the care/harm foundation prioritizes “harming others” over “harming the self” or that the sanctity/degradation foundation prioritizes “offending the self” over “offending others” in the process. This research—together with prior third-person effect research—filled this void by providing statistical evidence for this differential prioritization that the two moral foundations entail, thus helping to clarify how exactly people make moral judgments based on intuition.

### 4.2. Implications for theorizing the third-person effect

Prior research mostly relied on the other-protection hypothesis to make sense of the robust positive correlation between perceived media harm on others and censorship support (see Section 1.3.1). The present research suggests that the other-protection hypothesis provides an incomplete explanation of the third-person effect because using media offensiveness harm perceptions reversed the third-person effect. A complete explanation would need to be able to reconcile the seemingly inconsistent results observed with media harm and offensiveness perceptions.

Moral foundations theory may provide such an explanation. According to the moral foundations explanation of the third-person effect, the care/harm foundation underlies the correlation between perceived media harm on others and censorship support, whereas the sanctity/degradation foundation underlies the correlation between perceived media offensiveness to the self and censorship support. However, we also note several caveats with this explanation.

First off, Zhang (2017) found that the sanctity/degradation instead of the care/harm foundation positively predicted support of censoring beer commercials. Similarly, prior research provided mixed results on whether paternalism—a measure of the motive to protect others—positively predicted the support of censoring a wide range of socially undesirable media content (McLeod et al., 2001; Paek et al., 2008). Third, this research found that perceived media offensiveness to the self was a stronger predictor than perceived media harm on others of censorship support even with media violence, a type of program closely related to harm. Thus, a potential challenge to the moral foundations explanation of the third-person effect is to explain why, compared with variables related to the care/harm foundation, variables related to the sanctity/degradation foundation appear to be more reliable predictors of censorship support.

### 4.3. Limitations

In this research, we only used sexual, alcoholic, and violent media content as test cases, and whether the current findings would hold with other socially undesirable media content remains unclear. Second, we focused on the notion of offensiveness following prior work (Leone, 2001; Reid et al., 2007), but being offended includes several different emotions including anger and disgust. How the perceived media effects on those discrete emotions would predict censorship support remains to be tested. Third, what is considered offensive differs across cultures (Fam and Waller, 2003), and it is thus important to replicate the current research in countries and regions other than the U.S. Doing so would allow us to assess the extent to which the psychological mechanisms underlying the third-person effect is universal.

## 5. Conclusion

In this research, we found that offensiveness and harm perceptions relate to censorship support differently. At least in the U.S., people are more likely to support censoring sexual, alcoholic, and violent media content when they perceive the media content in question to harm other viewers more or to offend themselves more. This finding confirms that the perceptions of harm and offensiveness are important to people’s sense of free speech, but why those two perceptions concern different targets (i.e., others vs. the self) when predicting censorship support remains to be further explored.

## Data availability statement

The raw data supporting the conclusions of this article will be made available by the authors, without undue reservation. This data can be found here: https://osf.io/m7kh2/?view_only=ff984ab30ca048afb7fa79aed42871da.

## Ethics statement

The studies involving humans were approved by The University of Hawaii Institutional Review Board. The studies were conducted in accordance with the local legislation and institutional requirements. The participants provided their written informed consent to participate in this study.

## Author contributions

JZ conceived and designed the study, analyzed the data, and wrote the manuscript.

## Funding

This work was supported by Sun Yat-sen University’s startup funding to JZ (17000-12230014).

## Conflict of interest

The author declares that the research was conducted in the absence of any commercial or financial relationships that could be construed as a potential conflict of interest.

## Publisher’s note

All claims expressed in this article are solely those of the authors and do not necessarily represent those of their affiliated organizations, or those of the publisher, the editors and the reviewers. Any product that may be evaluated in this article, or claim that may be made by its manufacturer, is not guaranteed or endorsed by the publisher.

## Footnotes

^1^PME3 = perceived media effects on others; PME1 = perceived media effects on the self.

^2^Despite Chung and Moon’s (2016) method not requiring this step (because PME1 and PME3 would be used as separate predictors), we nevertheless performed that verification to mimic the condition in which prior research tested the third-person effect.

^3^To ensure that the prompt did not bias respondents’ responses responses to measures of offensiveness perceptions, we visually examined the distribution of all 12 relevant variables [i.e., 3 types of media content × 2 targets (self vs. others) × 2 studies]. Only two distributions showed clear signs of being negatively skewed (i.e., with many large values).

^4^We used the parameters of the model on alcoholic media content for power analysis because it yielded a non-significant comparison between perceived media offensiveness to the self and that to others with the Bonferroni correction.
