# Supplementary material for: Perceived offensiveness to the self, not that to others, is a robust positive predictor of support of censoring sexual, alcoholic, and violent media content
Source: Front Psychol. 2023 Aug 29;14:1159014. doi: 10.3389/fpsyg.2023.1159014 (PMC10495590; doi:10.3389/fpsyg.2023.1159014)
Supplement: Supplementary file 1 [file Data_Sheet_1.docx]

Supplementary Material

Perceived Offensiveness to the Self, Not That to Others, is a Robust Positive Predictor of Support of Censoring Sexual, Alcoholic, and Violent Media Content

Jinguang Zhang*

*** Correspondence:** Jinguang Zhang: zhangjg29@mail.sysu.edu.cn

# Regression diagnostics

## Study 1

We first checked whether the regression models suffered from collinearity, and they did not. All VIFs were smaller than 2 (well below 10, the conventional threshold that indicates the existence of collinearity). We then performed the Breusch-Pagan test to check heteroscedasticity and the Shapiro-Wilk test to check residual normality for the three OLS regression models performed in Study 1. For the Breusch-Pagan test, none of the results were significant at α= 0.05, providing no evidence of the presence of heteroscedasticity. The results of the Shapiro-Wilk test were significant, suggesting deviation in the residuals from normality (despite that the skewness values of the variables in the regression models were within acceptable ranges; see main text). We consulted multiple online sources on this issue, and the consensus appears to be that the normality of residuals is a relatively weak assumption that OLS regression is robust to with reasonably large samples. The sources are:

<https://stats.stackexchange.com/questions/272863/non-normality-in-multiple-linear-regression>

<https://stats.stackexchange.com/questions/616465/cut-off-value-of-1-5-for-skewness-and-kurtosis-tabachnick-fidell>

<https://stats.stackexchange.com/questions/149226/does-linear-regression-assume-all-variables-predictors-and-response-to-be-mult>

We also ran simulations to check the extent to which non-normality in predictor variables and/or in residuals would affect parameter estimations and statistical inferences. The short answer is that either form of non-normality tends to have minimum impact on parameter estimations and statistical inferences with a sample of N = 500, and the sample sizes of the two studies were both over 500. See https://osf.io/m7kh2/?view_only=50fdd19e8ac14efb9e776e7cc68b47b6 for the R code of simulation.

We also checked for data points of large influence and leverage (about 4-5 per variable), and dropping those data points did not change statistical conclusions of our OLS regression models.

## Study 2

As in Study 1, we first checked whether the regression models suffered from collinearity, and they did not. All VIFs were smaller than 2 (well below 10, the conventional threshold that indicates the existence of collinearity). We then performed the Breusch-Pagan test to check heteroscedasticity for the three OLS regression models performed in Study 2. The results indicated the presence of heteroscedasticity, and we thus re-ran the OLS regression models with robust standard errors. Doing so did not change statistical conclusions of our models except that perceived media harm on others no longer predicted support of censoring pornography. The Shapiro-Wilk test indicated deviation in the residuals from normality (despite that the skewness values of the variables in the regression models were within acceptable ranges; see main text). However, given the considerations above, we took no action and left the analyses as they were.

We also identified data points of potential high influence and/or leverage (about 3 per model). Excluding those data points did not affect results of our OLS regression models.

# Supplementary Tables

**Table S1**

***Summary of Measures of Presumed Media Effects in Studies Included in Chung and Moon’s (2016) Analysis and Those that Invoked the Other-Protection Hypothesis***

| **Authors (year)** | **Media Content** | **Measures of Presumed Media Effects** |
| --- | --- | --- |
| **Eveland et al. (1999)** | **Violent and misogynic rap and death metal lyrics** | **Respondents were asked to “estimate how influenced they believed themselves and 10 groups of people would be by ‘listening to songs with this type of lyrics’” (p. 284)** |
| **Gunther (1995)** | **Pornography** | **Questions about respondents’ “perceptions of the effects of such content on” their own and other people’s 1) “moral values concerning sex” and 2) “attitudes toward the opposite sex” (p. 29)** |
| **Gunther & Hwa (1996)** | **Sex and violence** | **“Respondents were asked to rate influence of such content on ‘themselves personally’… and on ‘the average Singaporean’” (p. 252)** |
| **Hoffner et al. (1999)** | **Violence** | **“Respondents rated the perceived effects of television violence on themselves and other adults” regarding 1) “viewing the world as a dangerous place,” 2) thinking aggression is acceptable,” and 3) “behaving aggressively” (p. 732)** |
| **Lee & Tamborini (2005)** | **Pornography** | **Respondents rated the extent to which they agreed that 1) “ponographic content on the Internet has a negative effect on your (other people’s) moral values concerning sex” and 2) “pornographic content on the Internet has a negative effect on your (other people’s) attitudes toward the opposite sex” (p. 300)** |
| **Lo et al. (2002)** | **Pornography** | **Respondents estimated “the likely negative effects of surfing pornographic websites on moral values, attitudes toward the opposite sex, sexual knowledge, sexual attitudes, and sexual behavior” on themselves and other students (p. 21)** |
| **McLeod et al. (1997)** | **Violent and misogynic rap lyrics** | **“Respondents were asked to estimate the effects of listening to songs with these types of lyrics on the knowledge, attitudes, and behaviors” of themselves and others (p. 161)** |
| **McLeod et al. (2001)** | **Violent and misogynic rap and death metal lyrics** | **Same as above** |
| **Neuwirth & Frederick (2002)** | **News article on prostitution** | **Respondents were asked to rate “the neighborhood prostitution story’s influence on what you (others) think” and “feel” (p. 122)** |
| **Neuwirth et al. (2002)** | **News article on the Mississippi state flag issue** | **“How much would a story about the state flag issue influence White state residents to have negative beliefs about African American state residents?” “How much do stories about the state flag issue influence African American state residents to feel negative emotions?” (p. 331)** |
| **Price et al. (1998)** | **Holocaust-denial advertisement** | **The questions asked “respondents to estimate how much influence the advertisement, if published, would have on their own (other people’s) attitudes toward the Holocaust” (p. 10)** |
| **Rojas et al. (1996)** | **Media in general, pornography, television violence** | **Respondents were asked to indicate how strongly they agreed or disagreed that “pornographic movies/magazines have a powerful effect on societal attitudes” and “my attitudes” (p. 171)** |
| **Salwen (1998)** | **Campaign messages** | **Respondents were asked about “their perceptions of how effective newspapers were in influencing other people’s (their own) opinions about the candidates” (p. 268)** |
| **Salwen & Dupagne (1999)** | **Violence; televised trials; and negative political advertising** | **Respondents rated how “powerful,” “persuasive,” “significant,” and “strong” the effects of the media content in question were on others and themselves (p. 531)** |
| **Shah et al. (1999)** | **Controversial products advertising; gambling services advertising** | **Respondents were asked to indicate how much they agreed or disagreed that “advertising for cigarettes, liquor, beer, casinos, or lotteries has a powerful effect on me and on many adults” (p. 249)** |
| **Shin & Kim (2011)** | **Alcohol product placement in youth-oriented movies** | **Respondents were asked to report their “perception of whether myself/others would be influenced to increase alcohol consumption by watching youth movies with scenes of the main actors drinking branded alcohol” (p. 424)** |

**Table S2**

*OLS Regression Results of Predicting Censorship Support from Perceived Media Offensiveness to the Self, That to Others, and Their Interaction Term. Study 1.*

|  | | | |
| --- | --- | --- | --- |
|  | Outcome variable: Support of censoring… | | |
|  |  | | |
|  | pornography | media portrayals of excessive drinking | media violence |
|  | | | |
| Perceived offensiveness to self | 0.477^***^ | 0.397^***^ | 0.339^***^ |
|  | (0.043) | (0.045) | (0.047) |
|  |  |  |  |
| Perceived offensiveness to others | 0.078^*^ | 0.127^***^ | 0.124^***^ |
|  | (0.040) | (0.043) | (0.044) |
|  |  |  |  |
|  |  |  |  |
| Respondents’ sex (0 = male, 1 = female) | 0.115^***^ | 0.079^**^ | 0.107^**^ |
|  | (0.040) | (0.039) | (0.042) |
|  |  |  |  |
| **Self × Others** | **0.006** | **-0.037** | **-0.003** |
|  | **(0.036)** | **(0.038)** | **(0.038)** |
|  |  |  |  |
|  |  |  |  |
| Constant | 0.013 | 0.021 | 0.001 |
|  | (0.039) | (0.042) | (0.043) |
|  |  |  |  |
|  | | | |
| Observations | 520 | 521 | 520 |
| R^2^ | 0.325 | 0.237 | 0.202 |
| Adjusted R^2^ | 0.320 | 0.231 | 0.196 |
|  | | | |

Note: Standard errors are in parentheses. ^*^*p* < .05; ^**^*p* < .01; ^***^*p* < 0.001

**Table S3**

*OLS Regression Results of Predicting Censorship Support from Perceived Media Offensiveness to the Self, That to Others, and Their Interaction Term. Study 2.*

|  | | | |
| --- | --- | --- | --- |
|  | Outcome variable: Support of censoring… | | |
|  |  | | |
|  | pornography | beer commercials | graphic violence |
|  | | | |
| Perceived offensiveness to self | 0.728^***^ | 0.610^***^ | 0.597^***^ |
|  | (0.030) | (0.036) | (0.035) |
|  |  |  |  |
| Perceived offensiveness to others | -0.023 | 0.126^***^ | 0.043 |
|  | (0.028) | (0.038) | (0.034) |
|  |  |  |  |
|  |  |  |  |
| Respondents’ sex (male = 0, female = 1) | 0.055^**^ | 0.066^**^ | 0.074^**^ |
|  | (0.027) | (0.027) | (0.030) |
|  |  |  |  |
| **Self × others** | **-0.003** | **-0.036** | **0.020** |
|  | **(0.025)** | **(0.027)** | **(0.028)** |
|  |  |  |  |
|  |  |  |  |
| Constant | 0.004 | 0.016 | -0.011 |
|  | (0.028) | (0.032) | (0.032) |
|  |  |  |  |
|  | | | |
| Observations | 713 | 712 | 710 |
| R^2^ | 0.543 | 0.471 | 0.414 |
| Adjusted R^2^ | 0.541 | 0.468 | 0.411 |
|  | | | |

Note: Standard errors are in parentheses. ^*^*p* < .05; ^**^*p* < .01; ^***^*p* < 0.001

**Table S4**

*OLS Regression Results of Predicting Censorship Support from Perceived Media Harm on Self, That on Others, and Their Interaction Term. Study 2.*

|  | | | |
| --- | --- | --- | --- |
|  | Outcome variable: Support of censoring… | | |
|  |  | | |
|  | pornography | beer commercials | graphic violence |
|  | | | |
| Perceived harm on self | 0.163^***^ | 0.212^***^ | 0.107^*^ |
|  | (0.058) | (0.057) | (0.061) |
|  |  |  |  |
| Perceived harm on others | 0.277^***^ | 0.381^***^ | 0.390^***^ |
|  | (0.053) | (0.047) | (0.052) |
|  |  |  |  |
|  |  |  |  |
| Respondents’ sex (0 = male, 1 = female) | 0.281^***^ | 0.140^***^ | 0.208^***^ |
|  | (0.033) | (0.031) | (0.032) |
|  |  |  |  |
| **Self × others** | **0.037** | **-0.002** | **-0.027** |
|  | **(0.038)** | **(0.038)** | **(0.037)** |
|  |  |  |  |
|  |  |  |  |
| Constant | -0.026 | -0.002 | 0.018 |
|  | (0.043) | (0.042) | (0.043) |
|  |  |  |  |
|  | | | |
| Observations | 713 | 711 | 711 |
| R^2^ | 0.267 | 0.318 | 0.268 |
| Adjusted R^2^ | 0.263 | 0.314 | 0.263 |
|  | | | |

Note: Standard errors are in parentheses. ^*^*p* < .05; ^**^*p* < .01; ^***^*p* < 0.001
